# Origin, tempo, and mode of the spread of DENV-4 Genotype IIB across the state of São Paulo, Brazil during the 2012-2013 outbreak

**DOI:** 10.1590/0074-02760180251

**Published:** 2019-01-07

**Authors:** Ayda Susana Ortiz-Baez, Marielton dos Passos Cunha, Danila Vedovello, Tatiana Elias Colombo, Maurício Lacerda Nogueira, Christian Julián Villabona-Arenas, Paolo Marinho de Andrade Zanotto

**Affiliations:** 1 Universidade de São Paulo, Instituto de Ciências Biomédicas, Departamento de Microbiologia, Laboratório de Evolução Molecular e Bioinformática, São Paulo, SP, Brasil; 2 Faculdade de Medicina de Jundiaí, Departamento de Pediatria, Laboratório de Infectologia Pediátrica, Jundiaí, SP, Brasil; 3 Faculdade de Medicina de São José do Rio Preto, São José do Rio Preto, SP, Brasil; 4 Université de Montpellier, Institut de Recherche pour le Développement, Montpellier, France; 5 Université de Montpellier, Institut de Biologie Computationnelle, Laboratoire d’Informatique, de Robotique et de Microélectronique de Montpellier, Montpellier, France

**Keywords:** Brazil, dengue virus type 4, genotype IIB, phylodynamics, São Paulo

## Abstract

**BACKGROUND:**

Dengue virus type 4 (DENV-4) was first reported in Brazil in 1982 and since then no more cases were detected again in Brazil until 2010, when the virus was reintroduced. Over the following years, the virus spread to several Brazilian states and resulted in about 1,400,000 dengue cases, in 2013. The largest number of cases were documented in the Southeast macro-region.

**OBJECTIVES:**

To determine the phylogeography of DENV-4 Genotype IIB strains isolated during the epidemics in 2012-2013 in São Paulo, Brazil, we aimed to contextualise the contribution of viruses sampled in different localities across the overall movement of DENV-4 in Brazil.

**METHODS:**

Based on the envelope gene sequences retrieved from GenBank, we employed a Bayesian phylogeographic approach to assess the spatiotemporal dynamics of DENV-4 Genotype IIB in São Paulo, Brazil.

**FINDINGS:**

The dispersal dynamics of DENV-4 Genotype IIB in Brazil indicated Rio de Janeiro and Mato Grosso states as the most likely routes toward São Paulo before the 2012-2013 outbreak. Likewise, Guarujá and São José do Rio Preto facilitated viral spread and transmission to other localities in the South and Southeast macro-regions in Brazil.

**CONCLUSIONS:**

The spread pattern of DENV-4 Genotype IIB strains across the country supports two independent introductions of the virus in São Paulo in a short period of time. Furthermore, São Paulo appears to have played a pivotal role in the dissemination of DENV-4 to other locations in Brazil.

Dengue virus (DENV) is a mosquito-borne pathogen that causes dengue, a disease that can range from dengue fever to severe forms of the disease.^1^ It is transmitted to humans during feeding of infected *Aedes* mosquitoes, mainly *Aedes aegypti*, which are widely distributed in the tropical and subtropical regions of the world.^1^ ^,^ ^2^ DENV is an enveloped virus of the genus *Flavivirus*, family *Flaviviridae*, which is also classified in four phylogenetically and antigenically distinct serotypes (DENV-1-4).^3^ The genetic diversity within each serotype is further subdivided into genotypes, which differ according to their spatiotemporal distribution.

The global spread of DENV has increased over the last two decades due to the increased transmission of DENV-4.^4^ DENV-4 is classified into four genotypes: (i) genotype I includes strains circulating in Southeast Asia; (ii) genotype II comprises American and Asian strains, and it is also subdivided into two clades named genotype IIA and IIB; (iii) genotype III comprises recent Thai strains; (iv) and the sylvatic genotype, which includes Malaysia strains. Previous phylogeographic analyses have demonstrated the presence of two distinct genotypes in Brazil.^5^ The virus was originally isolated in 1981-1982 in the state of Roraima and belonged to genotype II. Genotype I was not circulating in the Americas until recent years when sporadic infections were reported in the Brazilian states of Amazonas and Bahia.^6^

In 2010, DENV-4 Genotype II either re-emerged or was re-introduced in the state of Roraima, a northern macro-region of the country, after 28 years of no record of this genotype in the region. Previous analyses revealed that DENV-4 strains circulating in Brazil appeared to originate from Northern South America.^5^ ^,^ ^7^ Since then, multiple DENV-4 outbreaks have been observed, which have caused the disease to spread throughout several states,^7^ ^,^ ^8^ making DENV-4 the predominant serotype during the outbreak of 2012-2013^9^ with the simultaneous circulation of the other three serotypes.^10^

The 2013 DENV-4 Genotype IIB epidemic caused the highest incidence of the disease in the country; over 1,400,000 cases were reported, including 6,777 severe cases,^11^ which were associated with an economic burden of US$ 1,228 million without adding the cost of prevention and outbreak control.^12^ Brazil is divided into five geographical macro-regions (North, Northeast, Centre-west, Southeast, South). In 2013, the southeast macro-region presented a majority of the cases (60%), making São Paulo the second-most affected state in the country. The high number of cases in São Paulo represent a risk of dengue transmission to other Brazilian regions given its economic and touristic importance and because it is also the most populous state with the largest city (São Paulo) in the country. Despite the importance of DENV-4, the origin and spread pattern of DENV-4 towards São Paulo remain unknown. At present, no phylogeographical studies of DENV-4 at the regional level and comprising samples from the Southeast have been reported. The present study describes the epidemic spreading process of DENV-4 toward the state of São Paulo during the 2012-2013 outbreak.

## MATERIALS AND METHODS

**Fig. 1:**
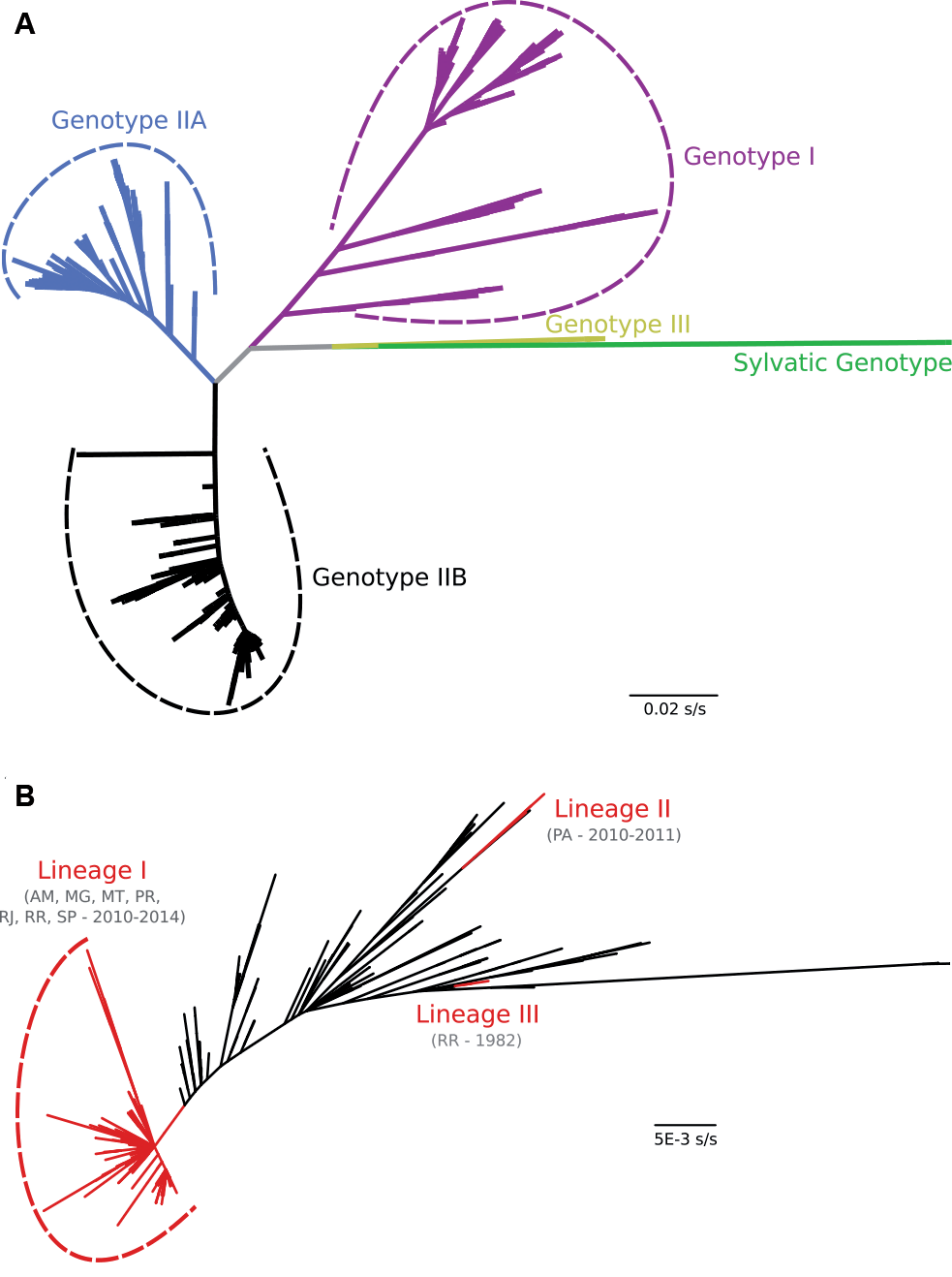
maximum likelihood phylogenetic tree of dengue virus type 4 (DENV-4) based on full-length envelope gene sequences (n = 1408). (A) DENV-4 genotypes. (B) Clustering of Brazilian sequences in lineages I-III. Location acronyms: Amazonas (AM); Minas Gerais (MG); Mato Grosso (MT); Pará (PA); Paraná (PR); Rio de Janeiro (RJ); Roraima (RR); São Paulo (SP).

*Sequences and dataset construction* - All available sequences were retrieved from GenBank (n = 1729), on September 2018. Data curation consisted in excluding problematic sequences (i.e., recombinant and chimeric sequences, clones, unverified sequences or without known sampling date, and partial envelope sequences). DENV-4 sequences were aligned with MAFFT v7.409 (“auto” settings), and the genomic region corresponding to the envelope gene was extracted. Next, we performed additional tests to identify and remove recombinant sequences with RDP4 Beta v4.96, using all available methods with their default settings. The statistical selection of the best-fit nucleotide substitution model was performed with jModelTest2. We reconstructed a ML tree based on the GTR+Γ4 model using the software FastTree v2.1 (http://www.microbesonline.org/fasttree/) under an exhaustive search, and computing local support values. Genotypes were identified based on phylogenetic grouping and literature.^13^ Sequences from São Paulo state fell into Genotype IIB. We therefore selected all sequences grouped into Genotype IIB and identified three Brazilian lineages (Fig. 1).

The final dataset resulted in 705 sequences [Supplementary data (Table I)]. Next, we created two subsets. To corroborate whether the re-emergent DENV-4 circulating in São Paulo and other Brazilian localities correspond to a single introduction, we built the first dataset (DAT-1). For DAT-1, we kept some Brazilian and international sequences from Genotype IIB, spanning a total of 20 countries [Supplementary data (Tables I-II)]. To reconstruct the spread of DENV-4 toward São Paulo, a second dataset (DAT-2) was constructed. For DAT-2, we kept only Brazilian sequences clustered with São Paulo sequences (Lineage I) [Fig. 1, Supplementary data (Tables I, III)].

To reduce sampling bias, sequences from each dataset were subsampled on the basis of their geographic sampling location. Therefore, for locations with more than three sequences, we used the UCLUST algorithm implemented in USEARCH v11 to identify and remove duplicate sequences regarding a centroid or representative sequence determined by the algorithm (Identity threshold = 1). Where it was possible, the same number of sequences (n = 5) was kept for each location using the Decrease Redundancy tool hosted at ExPASY (https://www.expasy.org/genomics), with a maximum similarity threshold of 99.5%. As a result, we obtained non-redundant representative sequences for DAT-1 (n = 58) and DAT-2 (n = 44).

**Fig. 2:**
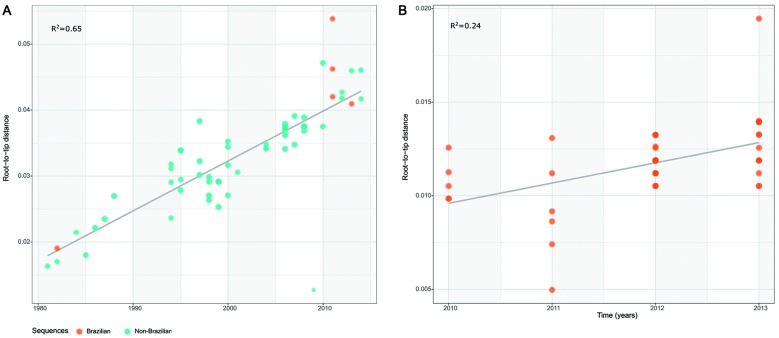
correlation between genetic divergence and sampling time from root-to-tip distances using dengue virus type 4 (DENV-4) sequences. Root-to-tip distances were inferred using a maximum likelihood phylogeny for DAT-1 (A) and DAT-2 (B).

*Molecular clock signal analysis* - The phylogenetic signal present in both datasets was assessed with TREE-PUZZLE v5.2 using the likelihood-mapping algorithm. We explored the temporal signal (i.e., molecular clock) and data quality with TempEst. Regression of root-to-tip genetic distance versus sampling was performed for each dataset (Fig. 2).

*Phylogeographic analysis* - Spatiotemporal pattern of DENV-4 Genotype IIB spread was reconstructed under a Bayesian framework as implemented in BEAST v1.10.1.^14^ For all the analyses a GTR+I+Γ4 model was selected as a substitution model using the AIC and BIC criteria implemented in jModelTest2. For each dataset, we tested strict and uncorrelated relaxed molecular clocks (log-normal distribution) in combination with population growth models: constant size, expansion, exponential and logistic growth. Phylogeography patterns and parameters were estimated running two independent Markov Chain Monte Carlo (MCMC) for 100 million states, and sampling every 10,000 states with 10% burn-in. Convergence and the effective sample size (ESS) > 200 were examined using Tracer v1.7.1 (http://beast.bio.ed.ac.uk/tracer). Likewise, the maximum clade credibility tree (MCC) was visualised and edited in FigTree v1.4.3 (http://tree.bio.ed.ac.uk/software/figtree/).

To overcome limitations regarding the low temporal signal in DAT-2, we specified an informative prior on the time of the most recent common ancestor (tMRCA). Hence, we set a normal distribution with a mean = 10.88 and standard deviation = 1.41 based on previous estimates of the Lineage I from DAT-1. From each MCC tree, we recorded the age of the tMRCA and its 95% Bayesian credible interval (HDP) (Table III).

For molecular clock and demographic model selection we used path sampling (PS) and stepping-stone (SS) approaches (Tables I-II), by running 100 path steps of 1 million iterations each. The demographic history of DENV-4 into Brazil was reconstructed under the Bayesian skyride coalescent model.

*Reconstruction of viral spread* - To reconstruct the viral diffusion of DENV-4 Genotype IIB within Brazil (DAT-2), an asymmetric discrete phylogeography model was used following a Bayesian stochastic search variable selection (BSSVS), with each locality used as a discrete state. The rates of diffusion were summarised using Bayes factor (BF > 3), and the spatiotemporal spread was visualised with SPREAD3.

## RESULTS

We reconstructed the spatiotemporal transmission dynamics of DENV-4 Genotype IIB, in order to gain insight into the origin of strains circulating in São Paulo during the 2012-2013 epidemic and investigate the relationships between these strains and those isolated from different Brazilian localities and the Americas.

The likelihood-mapping analysis suggested the presence of variable phylogenetic signal in our datasets. Approximately 74.8% of the quartets corresponded to fully resolved trees for DAT-1, whereas less than 50% were estimated for DAT-2. The high proportion of unresolved quarters (centre of the triangle) certainly might be associated with limitations associated with dataset size and the high similarity of DENV-4 sequences in DAT-2. No evidence of recombination was detected with any method.

Root-to-tips distances identified a moderate and low temporal signal in the data (*r* ^2^ = 0.65 for DAT-1, and *r* ^2^ = 0.24 for DAT-2) (Fig. 2). Therefore, we performed the analyses under a molecular clock assumption in combination with coalescent models (Tables I-II). For both datasets, the best-fit model involved a strict molecular clock. Nonetheless, for DAT-1 the exponential growth model outperformed the other models tested, while for DAT-2 the results suggest stronger support for a logistic growth model.

The overall evolutionary rate for DAT-1 was 7.87 E-4 substitutions/site/year [95% high probability densities (HPD): 76.51 E-4, 9.33 E-4]. Conversely, a relatively faster estimate of the substitution rate was observed for Brazilian sequences in DAT-2 (Table III).

**Fig. 3:**
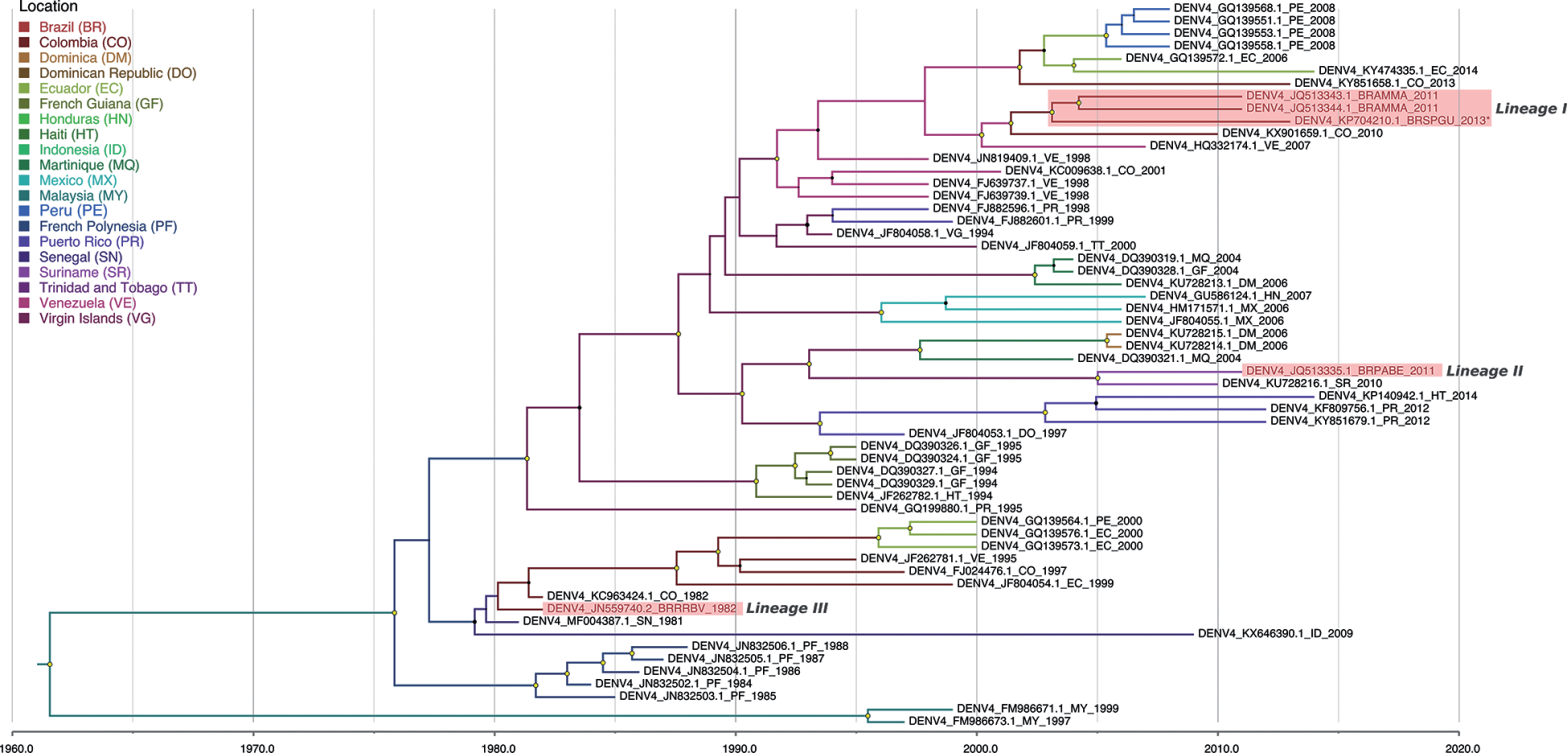
bayesian discrete phylogeography of genotype IIB dengue virus type 4 (DENV-4) strains (DAT-1). Maximum clade credibility (MCC) phylogeny inferred using envelope sequences. Brazilian sequences are grouped in three lineages designated as Lineage I-III. The DENV-4 strain from São Paulo state is indicated with an asterisk. The size of node circle is proportionate to the posterior probability of the node. Nodes with yellow circles have a support > 0.9. Branches are coloured according to the most probably ancestral lineage location.

Brazilian sequences analysed in this study followed three main routes for Genotype IIB into Brazil, designated as Lineages I-III (Fig. 3). Chronologically, the first event describes the introduction of DENV-4 into Roraima state, probably from Senegal (location state probability = 0.42). The second one describes the importation of DENV-4 into Pará state from the Caribbean (Suriname) (location state probability = 0.43), and the third one supports a more recent introduction of the virus into Roraima probably from Colombia (location state probability = 0.51). The lineage associated with the 2012-2013 outbreak in São Paulo (Lineage I) descended from this Colombian ancestor (Fig. 3). For the full dataset, Brazilian sequences clustered as Lineage I (Fig. 1) were used to build the DAT-2.

**TABLE I:** Model comparison of molecular clock and demographic growth models through path sampling (PS) and stepping stone (SS) methods for DAT-1. Bold numbers indicate the best fitting model

Demographic growth model	Relaxed molecular clock		Strict molecular clock	
	PS	SS	PS	SS
Constant	-5977.56	-5977.80	-5976.75	-5976.90
Exponential	-5968.79	-5968.73	-5965.7	-5965.94
Logistic	-5970.68	-5970.95	-5967.92	-5968.51
Expansional	-6075.05	-6075.04	-6071.88	-6071.98
Skyride	-5976.13	-5976.21	-5974.05	-5974.06

**TABLE II:** Model exploration of molecular clock and demographic growth models through path sampling (PS) and stepping stone (SS) methods for DAT-2. Bold numbers indicate the best fitting model

Demographic growth model	Relaxed molecular clock		Strict molecular clock	
	PS	SS	PS	SS
Constant	-3560.33	-3560.70	-3561.01	-3561.09
Exponential	-3553.47	-3553.99	-3552.04	-3552.38
Logistic	-3551.38	-3551.79	-3548.61	-3548.87
Expansional	-3562.04	-3562.22	-3566.09	-3566.23
Skyride	-3553.01	-3553.03	-3550.51	-3550.71

**TABLE III:** Estimation of evolutionary rate and time of the most recent common ancestor (TMRCA) for dengue virus type 4 (DENV-4) sequences

Clade	Mean TMRCA	Lower HPD	Upper HPD	Rate of evolution (s/s/y)
Genotype IIB*	1961.25	1953.01	1975.29	7.87 E-4 (6.51 E-4, 9.33 E-4)
Lineage I**	2007.91	2005.98	2009.49	2.50 E-3 (1.26 E-3, 3.90 E-3)

Datasets are represented as: *DAT-1; **DAT-2; HPD: high probability densities.

Once we identified the origin of the Brazilian strains circulating in São Paulo and neighbour states, we established the spread pattern of DENV-4 strains across the country until reach São Paulo, state. To deal with the lack of enough temporal signal in DAT-2, as suggested by the weak positive association between genetic divergence and sampling time (*r* ^*2*^ = 0.24) (Fig. 2B), we used a prior for the tMRCA based on previous estimates for Lineage I, from DAT-1.

**Fig. 4:**
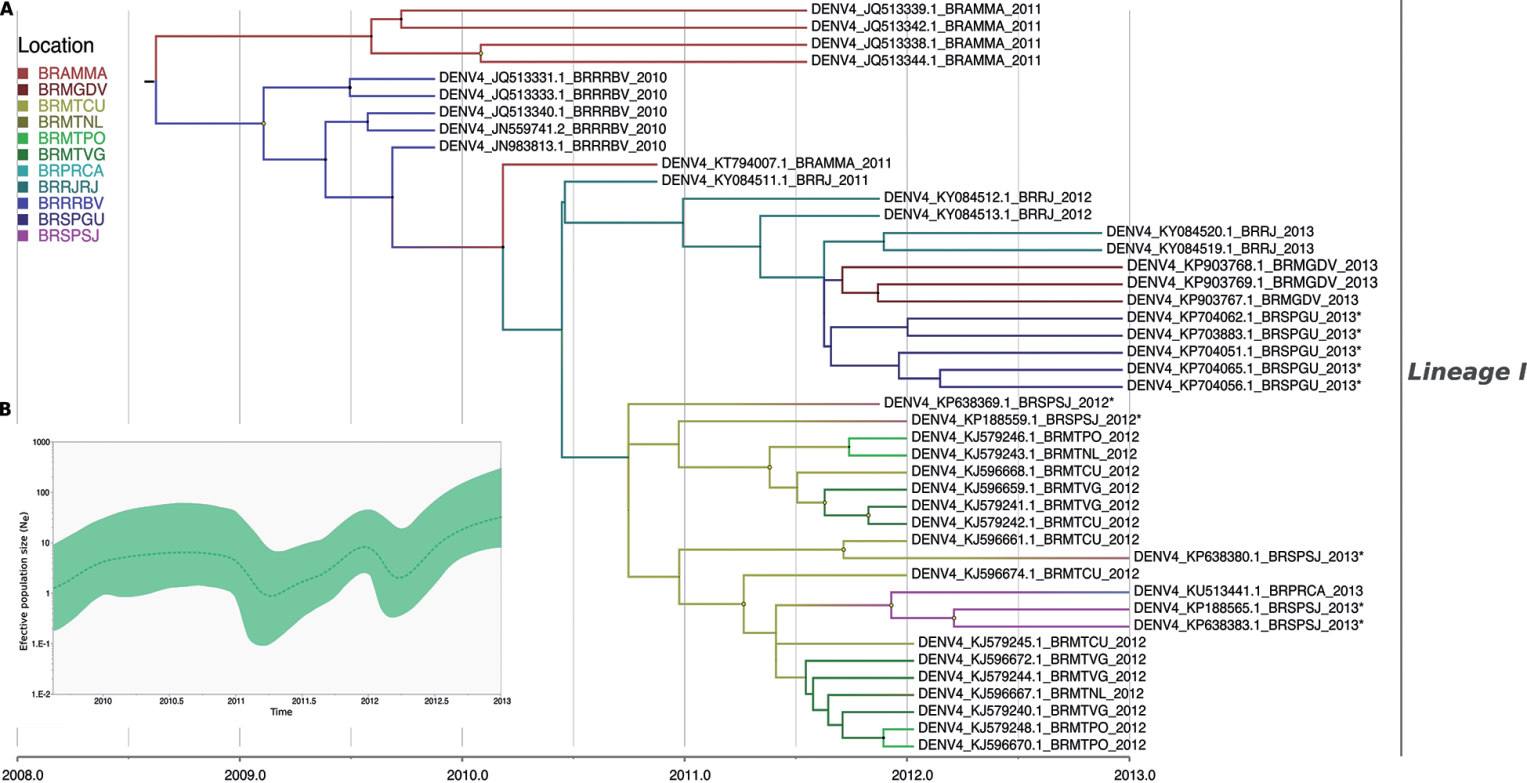
bayesian discrete phylogeography of Brazilian dengue virus type 4 (DENV-4) strains (DAT-2). (A) Maximum clade credibility (MCC) phylogeny inferred using envelope sequences. The designated lineage identity in Fig. 2 was maintained. DENV-4 strains from São Paulo state are indicated with asterisks. The size of node circle is proportionate to the posterior probability of the node. Nodes with yellow circles have a support > 0.9. Branches are coloured according to the most probable location of the ancestral lineage. (B) Bayesian skyride reconstruction showing population size fluctuations of DENV-4 in Brazil. The dotted dash line represents the median estimate and the shaded area shows the 95% credibility interval. Location acronyms: Amazonas, Manaus (BRAMMA); Minas Gerais, Divinópolis (BRMGDV); Mato Grosso, Cuiabá (BRMTCU); Mato Grosso, Nossa Senhora do Livramento (BRMTNL); Mato Grosso, Poconé (BRMTPO); Mato Grosso, Várzea Grande (BRMTVG); Paraná, Cambé (BRPRCA); Rio de Janeiro, Rio de Janeiro (BRRJRJ); Roraima, Boa Vista (BRRRBV); São Paulo, Guarujá (BRSPGU); São Paulo, São José do Rio Preto (BRSPSJ).

**Fig. 5:**
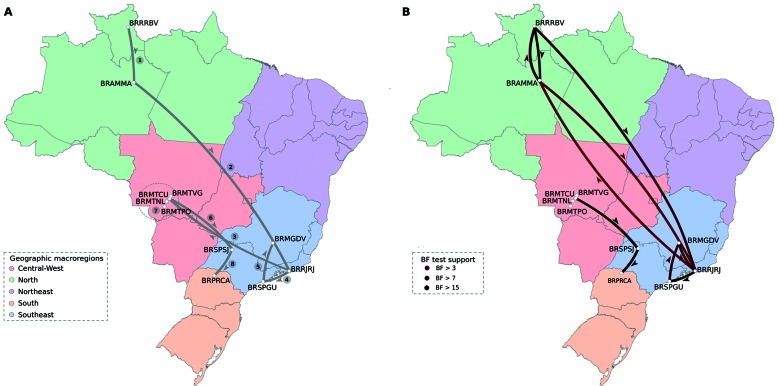
phylogeographic diffusion of dengue virus type 4 (DENV-4) across Brazil (DAT-2). (A) Spatial projection of the MCC tree. The grey lines represent branches connecting geographic locations. Numbers allow identifying the temporal order of diffusion events. The dotted dashed circle indicates the presence of several diffusion paths within Mato Grosso state, which are not exhibited here. (B) Spatial visualisation of significant non-zero rates using Bayes factor test (BF > 3). The line colour indicates the statistical support for each rate, weak (bright red) and strong (dark red). Arrows next to lines with upward and downward curvatures depict westward and eastward movements. Brazilian states are coloured by geographic macro-region. Location acronyms: Amazonas, Manaus (BRAMMA); Minas Gerais, Divinópolis (BRMGDV); Mato Grosso, Cuiabá (BRMTCU); Mato Grosso, Nossa Senhora do Livramento (BRMTNL); Mato Grosso, Poconé (BRMTPO); Mato Grosso, Várzea Grande (BRMTVG); Paraná, Cambé (BRPRCA); Rio de Janeiro, Rio de Janeiro (BRRJRJ); Roraima, Boa Vista (BRRRBV); São Paulo, Guarujá (BRSPGU); São Paulo, São José do Rio Preto (BRSPSJ).

Taken together, our results revealed that the virus circulating in São Paulo corresponded to the lineage most widely dispersed throughout Brazil (Figs 4-5).

Demographic history of DENV-4 in Brazil was characterised by fluctuations in population size through time. Thus, we observed an overall increasing trend disrupted by short periods of declination in population growth (Fig. 4B).

We recovered well-supported transition rates for most branches displayed in the MCC tree (Fig. 5). We found that strains from Guarujá, São Paulo were closely related to strains circulating in Minas Gerais along with strains from Rio de Janeiro. Noteworthy, the Bayesian reconstruction of the ancestral location revealed that the virus was dispersed from the North macro-region into Rio de Janeiro, and from Rio de Janeiro toward Guarujá (location state probability = 0.65) (Figs 4-5). Likewise, we identified well-supported rates (BF = 19.23) between both locations (Table IV). In addition, we identified to Guarujá as the most probably source of viral dissemination towards the Minas Gerais state (BF = 8.30). A second introduction of DENV-4 into São Paulo state was strongly supported from Cuiabá, Mato Grosso state to São José do Rio Preto, São Paulo (BF = 20.43). Viral movements among Brazilian localities were also observed in São José do Rio Preto into Paraná state (BF = 19.36) (Figs 4-5). We did not find statistical evidence supporting diffusion of DENV-4 between Guarujá and São José do Rio Preto in São Paulo (Fig. 5, Table IV).

## DISCUSSION

After the re-emergence of DENV-4 in Brazil, the spread of the virus across several Brazilian locations became a public health threat, reaching an unprecedented record of cases in the country.^15^ From 2011 to 2014 the national surveillance system reported more than 4.38 million suspected cases of dengue in Brazil, representing 61.7% of dengue cases in the Americas.^16^ Compared to previous years, the highest number of dengue cases was reported in 2013 with the re-emergence of DENV-4 associated with co-circulation of other three serotypes in Brazil.^9^ ^,^ ^10^ In the same year in São Paulo, 208,914 cases were reported, the highest number ever recorded.^11^ Moreover, previous studies have suggested the role of air traffic in the spatial diffusion of DENV toward Brazil, indicating that the virus could be moving quite rapidly in Brazil.^17^ Here, we incorporated spatiotemporal information in a phylogeography analysis to elucidate the origin and dispersal of DENV-4 in São Paulo, Brazil, which ultimately provided us some insights into the epidemiological setting of the virus. By including additional sequences from several localities, we recapitulated the phylogeography of DENV-4 Genotype IIB in Brazil (Fig. 3). We also obtained an estimate for the tMRCA for this genotype (1961) and added evidence that this virus has been circulating in Central and South America approximately 30 to 40 years ago.^5^ ^,^ ^7^ ^,^ ^18^ ^,^ ^19^ The overall evolutionary rate of 7.87E-4 subs/site/year agrees with similar results previously described for DENV-4^18^ ^,^ ^20^ and for other flaviviruses.^18^ ^,^ ^21^

**TABLE IV:** Estimated asymmetric rates of dengue virus type 4 (DENV-4) dispersal among locations. The table summarise transition rates with Bayes Factors (BF) support > 3

From	To	BF
BRMTCU	BRSPSJ	20.43990347
BRSPSJ	BRPRCA	19.3698586
BRRJRJ	BRSPGU	19.23297853
BRRRBV	BRAMMA	15.11944549
BRMTCU	BRMTVG	11.77424878
BRMTVG	BRMTCU	11.76894369
BRMTCU	BRMTPO	11.53294972
BRMTVG	BRMTPO	8.434459556
BRSPGU	BRMGDV	8.303935617
BRRRBV	BRRJRJ	7.930740464
BRRJRJ	BRMGDV	7.468288641
BRMTVG	BRMTNL	7.279020053
BRMTCU	BRMTNL	6.425809693
BRAMMA	BRRRBV	5.508072578
BRMTPO	BRMTNL	5.170309766
BRMTVG	BRSPSJ	3.904129255
BRAMMA	BRRJRJ	3.619004538
BRRJRJ	BRAMMA	3.453770397

Location acronyms: Amazonas, Manaus (BRAMMA); Minas Gerais, Divinópolis (BRMGDV); Mato Grosso, Cuiabá (BRMTCU); Mato Grosso, Nossa Senhora do Livramento (BRMTNL); Mato Grosso, Poconé (BRMTPO); Mato Grosso, Várzea Grande (BRMTVG); Paraná, Cambé (BRPRCA); Rio de Janeiro, Rio de Janeiro (BRRJRJ); Roraima, Boa Vista (BRRRBV); São Paulo, Guarujá (BRSPGU); São Paulo, São José do Rio Preto (BRSPSJ).

Evidence for the circulation of DENV-4 in Espírito Santo, Goiás, Mato Grosso do Sul and Minas Gerais between 2011 and 2013 has been published,^22^ ^,^ ^23^ ^,^ ^24^ thus suggesting the wide dissemination of the virus across the Centre-West and Southeast macro-regions. However, complete envelope sequences from these locations were not available to be included in our analyses.

Although there is evidence for the circulation of genotype I in Brazil,^6^ ^,^ ^25^ all samples included from São Paulo and other Brazilian localities belonged to genotype IIB. Similar to a recently published study, we identified multiple introductions of DENV-4 into Brazil.^5^ However, the virus circulating in São Paulo corresponded to the most broadly distributed and well-established lineage in Brazil, which also reached other states such as Amazonas, Espírito Santo, Mato Grosso, Rio de Janeiro, Rio Grande do Sul and Roraima.^5^ ^,^ ^23^ ^,^ ^26^ Remarkably, due to its equatorial position, Northern South American countries (e.g., Colombia, Guyana, Venezuela) have DENV activity during the entire year and seem to act as hubs of accumulation of viral genetic diversity and a common route for the traffic of viral pathogens to (e.g., DENV, CHIKV) and from (e.g., ZIKV) Brazil.^17^ ^,^ ^19^ ^,^ ^27^ ^,^ ^28^ Previous studies have demonstrated the re-emergence or re-introduction of DENV-4 in Roraima state and hypothesised its cryptic or imperceptible circulation before its detection.^5^ ^,^ ^7^

The phylogeographic reconstruction revealed that the virus was imported into São Paulo through two independent events in 2011, suggesting that continuous introductions are responsible for the maintenance of the virus in the state (Figs 4-5). The key role of Rio de Janeiro in the dissemination of DENV has been strongly suggested in previous studies.^26^ ^,^ ^29^ Nonetheless, little it is known about the importance of Mato Grosso in the maintenance and diffusion of DENV in Brazil.^22^ ^,^ ^30^

Our findings also support the notion that São Paulo state played an important role in the DENV-4 Genotype IIB spread to other Brazilian locations such as Divinópolis, Minas Gerais state and Cambé, Paraná (Figs 4-5). São Paulo state is characterised by an important commercial activity along a specialised service sector in Brazil. São José do Rio Preto is the main socio-economic hub in the west of the state of São Paulo that interconnects the Centre-West states to the east of the state of São Paulo. Conversely, Guarujá is an important Atlantic shore balneary and tourist destination. Together, the movement of viraemic travellers or infected vectors may promote the spread of the virus to other locations, and ultimately pose a significant risk to the dissemination of DENV to São Paulo city, the most densely populated metropolis of Brazil. Therefore, the geographic expansion of the virus highlighted the role of São Paulo as an important traffic centre of virus infection towards several localities in Brazil (Fig. 5). Reconstruction of the demographic history of DENV-4 could resemble the underlying dynamics of virus spread in Brazil. Increases in the effective population size suggest that the virus expanded geographically into new locations involving immunologically naïve populations. Once the susceptible hosts become unavailable, it is observed a decrease in the effective population size. This process, as shown in the Fig. 4B, is cyclical, suggesting continuous introductions of DENV-4 in different Brazilian localities.

In conclusion, the re-emergence of DENV-4 after approximately three decades in Brazil led to an unprecedented epidemic in the country. After virus entry, it spread along different routes. In this study, we reported that the circulation of DENV-4 Genotype IIB in São Paulo is the result of the expansion of the virus across the country. Our results also suggested two ancestral geographic origins as the most probable sources of DENV-4 in São Paulo. Our study emphasises the importance of implementing timely surveillance strategies across the country to reconstruct the dispersion pattern of DENV in the Brazilian territory.
