# Supplementary material for: Origin, tempo, and mode of the spread of DENV-4 Genotype IIB across the state of São Paulo, Brazil during the 2012-2013 outbreak
Source: Mem Inst Oswaldo Cruz. 2019 Jan 7;114:e180251. doi: 10.1590/0074-02760180251 (PMC6333047; doi:10.1590/0074-02760180251)
Supplement: Supplementary file 1 [file 1678-8060-mioc-114-e180251-s.pdf]

TABLE I

Dengue virus type 4 (DENV-4) strains included in this study for phylogeographic analyses. Countries and states are named using the ISO 3166-1 (alpha-2) code

Serotype	Accession code	Location	year	DAT-1	DAT-2
DENV-4	FM986673.1	MY	1997	yes	no
DENV-4	FM986672.1	MY	1997	no	no
DENV-4	FM986671.1	MY	1999	yes	no
DENV-4	FM986670.1	MY	1999	no	no
DENV-4	FM986669.1	MY	1999	no	no
DENV-4	FM986668.1	MY	1999	no	no
DENV-4	FM986667.1	MY	1999	no	no
DENV-4	FM986666.1	MY	1999	no	no
DENV-4	FM986665.1	MY	1999	no	no
DENV-4	FM986664.1	MY	1999	no	no
DENV-4	JN832506.1	PF	1988	yes	no
DENV-4	JN832505.1	PF	1987	yes	no
DENV-4	JN832504.1	PF	1986	yes	no
DENV-4	JN832503.1	PF	1985	yes	no
DENV-4	JN832501.1	PF	1983	no	no
DENV-4	JN832502.1	PF	1984	yes	no
DENV-4	KX646390.1	ID	2009	yes	no
DENV-4	MF004387.1	SN	1981	yes	no
DENV-4	JF804059.1	TT	2000	yes	no
DENV-4	JF804058.1	VG	1994	yes	no
DENV-4	MG895330.1	BR	2013	no	no
DENV-4	FJ226067.1	PR	1994	no	no
DENV-4	GQ252675.1	PR	1995	no	no
DENV-4	FJ850057.1	PR	1995	no	no
DENV-4	GQ199880.1	PR	1995	yes	no
DENV-4	GQ199879.1	PR	1994	no	no
DENV-4	FJ810417.1	PR	1995	no	no
DENV-4	FJ024476.1	CO	1997	yes	no
DENV-4	JF804052.1	CO	2006	no	no
DENV-4	GQ139573.1	EC	2000	yes	no
DENV-4	GQ139564.1	PE	2000	yes	no
DENV-4	GQ139576.1	EC	2000	yes	no
DENV-4	GQ139575.1	EC	2000	no	no
DENV-4	GQ139574.1	EC	2000	no	no
DENV-4	GQ139577.1	EC	2000	no	no
DENV-4	JF262781.1	VE	1995	yes	no
DENV-4	JF804054.1	EC	1999	yes	no
DENV-4	GU289913.1	CO	1982	no	no
DENV-4	JQ247980.1	BR	2009	no	no
DENV-4	KC963424.1	CO	1982	yes	no
DENV-4	JN559740.2	BRRRBV	1982	yes	no
DENV-4	KC963425.1	BR	1982	no	no
DENV-4	JQ513334.1	BRPASA	2010	no	no
DENV-4	JQ513335.1	BRPABE	2011	yes	no
DENV-4	JQ513337.1	BRPABE	2011	no	no
DENV-4	JQ513336.1	BRPABE	2011	no	no
DENV-4	KU728216.1	SR	2010	yes	no
DENV-4	JF804053.1	DO	1997	yes	no
DENV-4	KP140942.1	HT	2014	yes	no
DENV-4	JX476036.1	PR	2004	no	no
DENV-4	JX476037.1	PR	2006	no	no
DENV-4	KF809755.1	PR	2012	no	no
DENV-4	KF809760.1	PR	2013	no	no
DENV-4	KF809756.1	PR	2012	yes	no
DENV-4	JX476032.1	PR	2010	no	no

CC BY

Serotype	Accession code	Location	year	DAT-1	DAT-2
DENV-4	KF809758.1	PR	2012	no	no
DENV-4	KF809757.1	PR	2012	no	no
DENV-4	JQ045566.1	PR	2010	no	no
DENV-4	KF809754.1	PR	2012	no	no
DENV-4	KY851679.1	PR	2012	yes	no
DENV-4	KF809762.1	PR	2013	no	no
DENV-4	JX476035.1	PR	2010	no	no
DENV-4	KF809761.1	PR	2013	no	no
DENV-4	KF809759.1	PR	2012	no	no
DENV-4	JX476034.1	PR	2010	no	no
DENV-4	JX476033.1	PR	2010	no	no
DENV-4	JQ045565.1	PR	2010	no	no
DENV-4	JQ513344.1	BRAMMA	2011	yes	yes
DENV-4	KY851658.1	CO	2013	yes	no
DENV-4	JQ513343.1	BRAMMA	2011	yes	no
DENV-4	JQ513342.1	BRAMMA	2011	no	yes
DENV-4	KC009632.1	CO	2005	no	no
DENV-4	GQ868584.1	CO	2004	no	no
DENV-4	KC009639.1	CO	2000	no	no
DENV-4	GQ868583.1	CO	2004	no	no
DENV-4	KC009636.1	CO	2005	no	no
DENV-4	KY851660.1	CO	2013	no	no
DENV-4	GQ139583.1	VE	2000	no	no
DENV-4	GQ139586.1	VE	2001	no	no
DENV-4	FJ850095.1	VE	2000	no	no
DENV-4	GQ139584.1	VE	2000	no	no
DENV-4	KX901658.1	CO	2010	no	no
DENV-4	KX901657.1	CO	2009	no	no
DENV-4	FJ882581.1	VE	2007	no	no
DENV-4	EU854300.1	VE	2007	no	no
DENV-4	FJ882588.1	VE	2007	no	no
DENV-4	FJ882582.1	VE	2007	no	no
DENV-4	FJ639773.1	VE	2001	no	no
DENV-4	GQ868645.1	VE	2007	no	no
DENV-4	GQ868644.1	VE	2007	no	no
DENV-4	GQ868643.1	VE	2007	no	no
DENV-4	FJ882591.1	VE	2007	no	no
DENV-4	FJ882590.1	VE	2007	no	no
DENV-4	FJ882583.1	VE	2007	no	no
DENV-4	GQ868642.1	VE	2007	no	no
DENV-4	EU854299.1	VE	2007	no	no
DENV-4	FJ882589.1	VE	2007	no	no
DENV-4	KX901659.1	CO	2010	yes	no
DENV-4	GQ199876.1	VE	2007	no	no
DENV-4	HQ332176.1	VE	2007	no	no
DENV-4	FJ882585.1	VE	2007	no	no
DENV-4	FJ882592.1	VE	2008	no	no
DENV-4	EU854301.1	VE	2007	no	no
DENV-4	KC009640.1	CO	2004	no	no
DENV-4	KC009635.1	CO	2004	no	no
DENV-4	GQ868585.1	CO	2005	no	no
DENV-4	GQ139589.1	VE	2005	no	no
DENV-4	HQ332174.1	VE	2007	yes	no
DENV-4	FJ882580.1	VE	2007	no	no
DENV-4	GQ139591.1	VE	2007	no	no
DENV-4	FJ182016.1	VE	2007	no	no
DENV-4	FJ882584.1	VE	2007	no	no
DENV-4	GQ139587.1	VE	2004	no	no
DENV-4	GQ139588.1	VE	2004	no	no
DENV-4	FJ182017.1	VE	2007	no	no

Serotype	Accession code	Location	year	DAT-1	DAT-2
DENV-4	HQ332173.1	VE	2007	no	no
DENV-4	HQ332172.1	VE	2007	no	no
DENV-4	JN819406.1	VE	2006	no	no
DENV-4	GQ139590.1	VE	2005	no	no
DENV-4	HQ332175.1	VE	2007	no	no
DENV-4	FJ882587.1	VE	2007	no	no
DENV-4	FJ882586.1	VE	2007	no	no
DENV-4	KY474335.1	EC	2014	yes	no
DENV-4	KX901661.1	CO	2015	no	no
DENV-4	KX901660.1	CO	2014	no	no
DENV-4	GQ139572.1	EC	2006	yes	no
DENV-4	JF967780.1	PE	2010	no	no
DENV-4	GQ139553.1	PE	2008	yes	no
DENV-4	GQ139552.1	PE	2008	no	no
DENV-4	GQ139551.1	PE	2008	yes	no
DENV-4	GQ139548.1	PE	2008	no	no
DENV-4	GQ139547.1	PE	2008	no	no
DENV-4	GQ139571.1	PE	2008	no	no
DENV-4	GQ139570.1	PE	2008	no	no
DENV-4	GQ139569.1	PE	2008	no	no
DENV-4	GQ139567.1	PE	2008	no	no
DENV-4	GQ139566.1	PE	2008	no	no
DENV-4	GQ139565.1	PE	2008	no	no
DENV-4	GQ139568.1	PE	2008	yes	no
DENV-4	GQ139563.1	PE	2006	no	no
DENV-4	GQ139562.1	PE	2007	no	no
DENV-4	GQ139549.1	PE	2008	no	no
DENV-4	GQ139555.1	PE	2008	no	no
DENV-4	GQ139554.1	PE	2008	no	no
DENV-4	GQ139550.1	PE	2008	no	no
DENV-4	GQ139560.1	PE	2008	no	no
DENV-4	GQ139559.1	PE	2008	no	no
DENV-4	GQ139557.1	PE	2008	no	no
DENV-4	GQ139556.1	PE	2008	no	no
DENV-4	GQ139558.1	PE	2008	yes	no
DENV-4	GQ139561.1	PE	2008	no	no
DENV-4	JQ513338.1	BRAMMA	2011	no	yes
DENV-4	KY851659.1	CO	2013	no	no
DENV-4	KP188565.1	BRSPSJ	2013	no	yes
DENV-4	JQ513339.1	BRAMMA	2011	no	yes
DENV-4	KY851661.1	BR	2013	no	no
DENV-4	KP903769.1	BRMGDV	2013	no	yes
DENV-4	KY851665.1	BR	2013	no	no
DENV-4	KY084512.1	BRRJ	2012	no	yes
DENV-4	KP188561.1	BRSPSJ	2012	no	no
DENV-4	KP638369.1	BRSPSJ	2012	no	yes
DENV-4	KP638368.1	BRSPSJ	2012	no	no
DENV-4	KP188557.1	BRSPSJ	2012	no	no
DENV-4	KP638365.1	BRSPSJ	2012	no	no
DENV-4	KP638357.1	BRSPSJ	2012	no	no
DENV-4	KP638356.1	BRSPSJ	2012	no	no
DENV-4	KP704194.1	BRSPGU	2013	no	no
DENV-4	KP704021.1	BRSPGU	2013	no	no
DENV-4	KP703996.1	BRSPGU	2013	no	no
DENV-4	KP703941.1	BRSPGU	2013	no	no
DENV-4	KP704184.1	BRSPGU	2013	no	no
DENV-4	KP704204.1	BRSPGU	2013	no	no
DENV-4	KP704165.1	BRSPGU	2013	no	no
DENV-4	KP704052.1	BRSPGU	2013	no	no
DENV-4	KP704144.1	BRSPGU	2013	no	no

Serotype	Accession code	Location	year	DAT-1	DAT-2
DENV-4	KP703999.1	BRSPGU	2013	no	no
DENV-4	KP704161.1	BRSPGU	2013	no	no
DENV-4	KP703929.1	BRSPGU	2013	no	no
DENV-4	KP704163.1	BRSPGU	2013	no	no
DENV-4	KP704148.1	BRSPGU	2013	no	no
DENV-4	KP703903.1	BRSPGU	2013	no	no
DENV-4	KP704199.1	BRSPGU	2013	no	no
DENV-4	KP704166.1	BRSPGU	2013	no	no
DENV-4	KP704050.1	BRSPGU	2013	no	no
DENV-4	KP703907.1	BRSPGU	2013	no	no
DENV-4	KP704151.1	BRSPGU	2013	no	no
DENV-4	KP703998.1	BRSPGU	2013	no	no
DENV-4	KP703967.1	BRSPGU	2013	no	no
DENV-4	KP704214.1	BRSPGU	2013	no	no
DENV-4	KP704191.1	BRSPGU	2013	no	no
DENV-4	KP704180.1	BRSPGU	2013	no	no
DENV-4	KP704176.1	BRSPGU	2013	no	no
DENV-4	KP704172.1	BRSPGU	2013	no	no
DENV-4	KP704157.1	BRSPGU	2013	no	no
DENV-4	KP704149.1	BRSPGU	2013	no	no
DENV-4	KP704112.1	BRSPGU	2013	no	no
DENV-4	KP704104.1	BRSPGU	2013	no	no
DENV-4	KP704096.1	BRSPGU	2013	no	no
DENV-4	KP704092.1	BRSPGU	2013	no	no
DENV-4	KP704079.1	BRSPGU	2013	no	no
DENV-4	KP704076.1	BRSPGU	2013	no	no
DENV-4	KP704071.1	BRSPGU	2013	no	no
DENV-4	KP704060.1	BRSPGU	2013	no	no
DENV-4	KP704059.1	BRSPGU	2013	no	no
DENV-4	KP704049.1	BRSPGU	2013	no	no
DENV-4	KP704042.1	BRSPGU	2013	no	no
DENV-4	KP704039.1	BRSPGU	2013	no	no
DENV-4	KP704038.1	BRSPGU	2013	no	no
DENV-4	KP704030.1	BRSPGU	2013	no	no
DENV-4	KP704015.1	BRSPGU	2013	no	no
DENV-4	KP704007.1	BRSPGU	2013	no	no
DENV-4	KP703991.1	BRSPGU	2013	no	no
DENV-4	KP703984.1	BRSPGU	2013	no	no
DENV-4	KP703918.1	BRSPGU	2013	no	no
DENV-4	KP703896.1	BRSPGU	2013	no	no
DENV-4	KP703886.1	BRSPGU	2013	no	no
DENV-4	KP703884.1	BRSPGU	2013	no	no
DENV-4	KP703873.1	BRSPGU	2013	no	no
DENV-4	KP704124.1	BRSPGU	2013	no	no
DENV-4	KP704110.1	BRSPGU	2013	no	no
DENV-4	KP704010.1	BRSPGU	2013	no	no
DENV-4	KP703983.1	BRSPGU	2013	no	no
DENV-4	KP703900.1	BRSPGU	2013	no	no
DENV-4	KP703966.1	BRSPGU	2013	no	no
DENV-4	KP703955.1	BRSPGU	2013	no	no
DENV-4	KP704175.1	BRSPGU	2013	no	no
DENV-4	KP704133.1	BRSPGU	2013	no	no
DENV-4	KP704121.1	BRSPGU	2013	no	no
DENV-4	KP704117.1	BRSPGU	2013	no	no
DENV-4	KP704085.1	BRSPGU	2013	no	no
DENV-4	KP704031.1	BRSPGU	2013	no	no
DENV-4	KP704026.1	BRSPGU	2013	no	no
DENV-4	KP704018.1	BRSPGU	2013	no	no
DENV-4	KP704008.1	BRSPGU	2013	no	no
DENV-4	KP703987.1	BRSPGU	2013	no	no

Serotype	Accession code	Location	year	DAT-1	DAT-2
DENV-4	KP703973.1	BRSPGU	2013	no	no
DENV-4	KP703971.1	BRSPGU	2013	no	no
DENV-4	KP703922.1	BRSPGU	2013	no	no
DENV-4	KP703920.1	BRSPGU	2013	no	no
DENV-4	KP703899.1	BRSPGU	2013	no	no
DENV-4	KP704109.1	BRSPGU	2013	no	no
DENV-4	KP704139.1	BRSPGU	2013	no	no
DENV-4	KP704137.1	BRSPGU	2013	no	no
DENV-4	KP704127.1	BRSPGU	2013	no	no
DENV-4	KP704093.1	BRSPGU	2013	no	no
DENV-4	KP704011.1	BRSPGU	2013	no	no
DENV-4	KP703962.1	BRSPGU	2013	no	no
DENV-4	KP704217.1	BRSPGU	2013	no	no
DENV-4	KP704215.1	BRSPGU	2013	no	no
DENV-4	KP704212.1	BRSPGU	2013	no	no
DENV-4	KP704203.1	BRSPGU	2013	no	no
DENV-4	KP704198.1	BRSPGU	2013	no	no
DENV-4	KP704197.1	BRSPGU	2013	no	no
DENV-4	KP703892.1	BRSPGU	2013	no	no
DENV-4	KP704169.1	BRSPGU	2013	no	no
DENV-4	KP704156.1	BRSPGU	2013	no	no
DENV-4	KP704162.1	BRSPGU	2013	no	no
DENV-4	KP704088.1	BRSPGU	2013	no	no
DENV-4	KP704130.1	BRSPGU	2013	no	no
DENV-4	KP704101.1	BRSPGU	2013	no	no
DENV-4	KP704099.1	BRSPGU	2013	no	no
DENV-4	KP704098.1	BRSPGU	2013	no	no
DENV-4	KP704025.1	BRSPGU	2013	no	no
DENV-4	KP703912.1	BRSPGU	2013	no	no
DENV-4	KP703894.1	BRSPGU	2013	no	no
DENV-4	KP704087.1	BRSPGU	2013	no	no
DENV-4	KP704083.1	BRSPGU	2013	no	no
DENV-4	KP704017.1	BRSPGU	2013	no	no
DENV-4	KP703931.1	BRSPGU	2013	no	no
DENV-4	KP703881.1	BRSPGU	2013	no	no
DENV-4	KP704055.1	BRSPGU	2013	no	no
DENV-4	KP703958.1	BRSPGU	2013	no	no
DENV-4	KP703948.1	BRSPGU	2013	no	no
DENV-4	KP703942.1	BRSPGU	2013	no	no
DENV-4	KP703927.1	BRSPGU	2013	no	no
DENV-4	KP703919.1	BRSPGU	2013	no	no
DENV-4	KP703913.1	BRSPGU	2013	no	no
DENV-4	KP703910.1	BRSPGU	2013	no	no
DENV-4	KP703905.1	BRSPGU	2013	no	no
DENV-4	KP703869.1	BRSPGU	2013	no	no
DENV-4	KP704216.1	BRSPGU	2013	no	no
DENV-4	KP704080.1	BRSPGU	2013	no	no
DENV-4	KP704013.1	BRSPGU	2013	no	no
DENV-4	KP704213.1	BRSPGU	2013	no	no
DENV-4	KP704211.1	BRSPGU	2013	no	no
DENV-4	KP704209.1	BRSPGU	2013	no	no
DENV-4	KP704208.1	BRSPGU	2013	no	no
DENV-4	KP704207.1	BRSPGU	2013	no	no
DENV-4	KP704202.1	BRSPGU	2013	no	no
DENV-4	KP704201.1	BRSPGU	2013	no	no
DENV-4	KP704200.1	BRSPGU	2013	no	no
DENV-4	KP704196.1	BRSPGU	2013	no	no
DENV-4	KP704185.1	BRSPGU	2013	no	no
DENV-4	KP704183.1	BRSPGU	2013	no	no
DENV-4	KP704179.1	BRSPGU	2013	no	no

Serotype	Accession code	Location	year	DAT-1	DAT-2
DENV-4	KP704177.1	BRSPGU	2013	no	no
DENV-4	KP704174.1	BRSPGU	2013	no	no
DENV-4	KP704173.1	BRSPGU	2013	no	no
DENV-4	KP704170.1	BRSPGU	2013	no	no
DENV-4	KP704168.1	BRSPGU	2013	no	no
DENV-4	KP704167.1	BRSPGU	2013	no	no
DENV-4	KP704164.1	BRSPGU	2013	no	no
DENV-4	KP704160.1	BRSPGU	2013	no	no
DENV-4	KP704158.1	BRSPGU	2013	no	no
DENV-4	KP704155.1	BRSPGU	2013	no	no
DENV-4	KP704154.1	BRSPGU	2013	no	no
DENV-4	KP704153.1	BRSPGU	2013	no	no
DENV-4	KP704152.1	BRSPGU	2013	no	no
DENV-4	KP704150.1	BRSPGU	2013	no	no
DENV-4	KP704147.1	BRSPGU	2013	no	no
DENV-4	KP704142.1	BRSPGU	2013	no	no
DENV-4	KP704140.1	BRSPGU	2013	no	no
DENV-4	KP704138.1	BRSPGU	2013	no	no
DENV-4	KP704136.1	BRSPGU	2013	no	no
DENV-4	KP704135.1	BRSPGU	2013	no	no
DENV-4	KP704132.1	BRSPGU	2013	no	no
DENV-4	KP704131.1	BRSPGU	2013	no	no
DENV-4	KP704129.1	BRSPGU	2013	no	no
DENV-4	KP704128.1	BRSPGU	2013	no	no
DENV-4	KP704125.1	BRSPGU	2013	no	no
DENV-4	KP704123.1	BRSPGU	2013	no	no
DENV-4	KP704120.1	BRSPGU	2013	no	no
DENV-4	KP704119.1	BRSPGU	2013	no	no
DENV-4	KP704114.1	BRSPGU	2013	no	no
DENV-4	KP704113.1	BRSPGU	2013	no	no
DENV-4	KP704111.1	BRSPGU	2013	no	no
DENV-4	KP704100.1	BRSPGU	2013	no	no
DENV-4	KP704097.1	BRSPGU	2013	no	no
DENV-4	KP704095.1	BRSPGU	2013	no	no
DENV-4	KP704094.1	BRSPGU	2013	no	no
DENV-4	KP704090.1	BRSPGU	2013	no	no
DENV-4	KP704089.1	BRSPGU	2013	no	no
DENV-4	KP704082.1	BRSPGU	2013	no	no
DENV-4	KP704078.1	BRSPGU	2013	no	no
DENV-4	KP704077.1	BRSPGU	2013	no	no
DENV-4	KP704073.1	BRSPGU	2013	no	no
DENV-4	KP704072.1	BRSPGU	2013	no	no
DENV-4	KP704069.1	BRSPGU	2013	no	no
DENV-4	KP704067.1	BRSPGU	2013	no	no
DENV-4	KP704061.1	BRSPGU	2013	no	no
DENV-4	KP704058.1	BRSPGU	2013	no	no
DENV-4	KP704053.1	BRSPGU	2013	no	no
DENV-4	KP704047.1	BRSPGU	2013	no	no
DENV-4	KP704046.1	BRSPGU	2013	no	no
DENV-4	KP704037.1	BRSPGU	2013	no	no
DENV-4	KP704036.1	BRSPGU	2013	no	no
DENV-4	KP704034.1	BRSPGU	2013	no	no
DENV-4	KP704032.1	BRSPGU	2013	no	no
DENV-4	KP704029.1	BRSPGU	2013	no	no
DENV-4	KP704028.1	BRSPGU	2013	no	no
DENV-4	KP704024.1	BRSPGU	2013	no	no
DENV-4	KP704023.1	BRSPGU	2013	no	no
DENV-4	KP704022.1	BRSPGU	2013	no	no
DENV-4	KP704016.1	BRSPGU	2013	no	no
DENV-4	KP704009.1	BRSPGU	2013	no	no

Serotype	Accession code	Location	year	DAT-1	DAT-2
DENV-4	KP704004.1	BRSPGU	2013	no	no
DENV-4	KP704002.1	BRSPGU	2013	no	no
DENV-4	KP703997.1	BRSPGU	2013	no	no
DENV-4	KP703994.1	BRSPGU	2013	no	no
DENV-4	KP703992.1	BRSPGU	2013	no	no
DENV-4	KP703990.1	BRSPGU	2013	no	no
DENV-4	KP703989.1	BRSPGU	2013	no	no
DENV-4	KP703986.1	BRSPGU	2013	no	no
DENV-4	KP703985.1	BRSPGU	2013	no	no
DENV-4	KP703978.1	BRSPGU	2013	no	no
DENV-4	KP703970.1	BRSPGU	2013	no	no
DENV-4	KP703964.1	BRSPGU	2013	no	no
DENV-4	KP703960.1	BRSPGU	2013	no	no
DENV-4	KP703959.1	BRSPGU	2013	no	no
DENV-4	KP703956.1	BRSPGU	2013	no	no
DENV-4	KP703954.1	BRSPGU	2013	no	no
DENV-4	KP703952.1	BRSPGU	2013	no	no
DENV-4	KP703951.1	BRSPGU	2013	no	no
DENV-4	KP703949.1	BRSPGU	2013	no	no
DENV-4	KP703947.1	BRSPGU	2013	no	no
DENV-4	KP703945.1	BRSPGU	2013	no	no
DENV-4	KP703944.1	BRSPGU	2013	no	no
DENV-4	KP703938.1	BRSPGU	2013	no	no
DENV-4	KP703937.1	BRSPGU	2013	no	no
DENV-4	KP703936.1	BRSPGU	2013	no	no
DENV-4	KP703932.1	BRSPGU	2013	no	no
DENV-4	KP703930.1	BRSPGU	2013	no	no
DENV-4	KP703928.1	BRSPGU	2013	no	no
DENV-4	KP703926.1	BRSPGU	2013	no	no
DENV-4	KP703924.1	BRSPGU	2013	no	no
DENV-4	KP703916.1	BRSPGU	2013	no	no
DENV-4	KP703914.1	BRSPGU	2013	no	no
DENV-4	KP703911.1	BRSPGU	2013	no	no
DENV-4	KP703909.1	BRSPGU	2013	no	no
DENV-4	KP703904.1	BRSPGU	2013	no	no
DENV-4	KP703902.1	BRSPGU	2013	no	no
DENV-4	KP703901.1	BRSPGU	2013	no	no
DENV-4	KP703893.1	BRSPGU	2013	no	no
DENV-4	KP703890.1	BRSPGU	2013	no	no
DENV-4	KP703889.1	BRSPGU	2013	no	no
DENV-4	KP703876.1	BRSPGU	2013	no	no
DENV-4	KP703872.1	BRSPGU	2013	no	no
DENV-4	KP703871.1	BRSPGU	2013	no	no
DENV-4	KP703870.1	BRSPGU	2013	no	no
DENV-4	KP703868.1	BRSPGU	2013	no	no
DENV-4	KP703867.1	BRSPGU	2013	no	no
DENV-4	KP703866.1	BRSPGU	2013	no	no
DENV-4	KP704195.1	BRSPGU	2013	no	no
DENV-4	KP704190.1	BRSPGU	2013	no	no
DENV-4	KP704122.1	BRSPGU	2013	no	no
DENV-4	KP704115.1	BRSPGU	2013	no	no
DENV-4	KP704006.1	BRSPGU	2013	no	no
DENV-4	KP703979.1	BRSPGU	2013	no	no
DENV-4	KP703957.1	BRSPGU	2013	no	no
DENV-4	KP704189.1	BRSPGU	2013	no	no
DENV-4	KP704186.1	BRSPGU	2013	no	no
DENV-4	KP703883.1	BRSPGU	2013	no	yes
DENV-4	KP703874.1	BRSPGU	2013	no	no
DENV-4	KP703975.1	BRSPGU	2013	no	no
DENV-4	KP703972.1	BRSPGU	2013	no	no

Serotype	Accession code	Location	year	DAT-1	DAT-2
DENV-4	KP703968.1	BRSPGU	2013	no	no
DENV-4	KP703977.1	BRSPGU	2013	no	no
DENV-4	KP704075.1	BRSPGU	2013	no	no
DENV-4	KP704051.1	BRSPGU	2013	no	yes
DENV-4	KP638380.1	BRSPSJ	2013	no	yes
DENV-4	KP188564.1	BRSPSJ	2013	no	no
DENV-4	KY851674.1	BR	2012	no	no
DENV-4	KP903767.1	BRMGDV	2013	no	yes
DENV-4	KY851752.1	BR	2013	no	no
DENV-4	KY851757.1	BR	2013	no	no
DENV-4	KY851678.1	BR	2013	no	no
DENV-4	KP638381.1	BRSPSJ	2013	no	no
DENV-4	KP704106.1	BRSPGU	2013	no	no
DENV-4	KJ596674.1	BRMTCU	2012	no	yes
DENV-4	KJ579244.1	BRMTVG	2012	no	yes
DENV-4	KJ579245.1	BRMTCU	2012	no	yes
DENV-4	KJ596662.1	BRMTCU	2012	no	no
DENV-4	KJ596666.1	BRMTCU	2012	no	no
DENV-4	KJ596672.1	BRMTVG	2012	no	yes
DENV-4	KJ579248.1	BRMTPO	2012	no	yes
DENV-4	KJ579240.1	BRMTVG	2012	no	yes
DENV-4	KJ596660.1	BRMTCU	2012	no	no
DENV-4	KJ596670.1	BRMTPO	2012	no	yes
DENV-4	KJ596664.1	BRMTCU	2012	no	no
DENV-4	KJ596667.1	BRMTNL	2012	no	yes
DENV-4	KJ596673.1	BRMTCU	2012	no	no
DENV-4	KJ596669.1	BRMTCU	2012	no	no
DENV-4	KJ579247.1	BRMTVG	2012	no	no
DENV-4	KJ596671.1	BRMTVG	2012	no	no
DENV-4	KP638385.1	BRSPSJ	2013	no	no
DENV-4	KP638372.1	BRSPSJ	2012	no	no
DENV-4	KP638383.1	BRSPSJ	2013	no	yes
DENV-4	KU513441.1	BRPRCA	2013	no	yes
DENV-4	KP638387.1	BRSPSJ	2013	no	no
DENV-4	KP638386.1	BRSPSJ	2013	no	no
DENV-4	KP638382.1	BRSPSJ	2013	no	no
DENV-4	KP188566.1	BRSPSJ	2013	no	no
DENV-4	KP188562.1	BRSPSJ	2012	no	no
DENV-4	KP188563.1	BRSPSJ	2013	no	no
DENV-4	KP638374.1	BRSPSJ	2013	no	no
DENV-4	KP638375.1	BRSPSJ	2013	no	no
DENV-4	KP638377.1	BRSPSJ	2014	no	no
DENV-4	KP638384.1	BRSPSJ	2013	no	no
DENV-4	KY084520.1	BRRJ	2013	no	yes
DENV-4	KJ596661.1	BRMTCU	2012	no	yes
DENV-4	JQ513340.1	BRRRBV	2010	no	yes
DENV-4	KY851753.1	BR	2013	no	no
DENV-4	KY851755.1	BR	2013	no	no
DENV-4	KY851756.1	BR	2013	no	no
DENV-4	KY851758.1	BR	2013	no	no
DENV-4	KY851749.1	BR	2012	no	no
DENV-4	KY851721.1	BR	2012	no	no
DENV-4	KJ596659.1	BRMTVG	2012	no	yes
DENV-4	KJ579243.1	BRMTNL	2012	no	yes
DENV-4	KJ596668.1	BRMTCU	2012	no	yes
DENV-4	KJ596658.1	BRMTCU	2012	no	no
DENV-4	KJ596665.1	BRMTCU	2012	no	no
DENV-4	KJ596663.1	BRMTCU	2012	no	no
DENV-4	KJ579246.1	BRMTPO	2012	no	yes
DENV-4	KJ579242.1	BRMTCU	2012	no	yes

Serotype	Accession code	Location	year	DAT-1	DAT-2
DENV-4	KJ579241.1	BRMTVG	2012	no	yes
DENV-4	KP638361.1	BRSPSJ	2012	no	no
DENV-4	KP903768.1	BRMGDV	2013	no	yes
DENV-4	KP704048.1	BRSPGU	2013	no	no
DENV-4	KP188559.1	BRSPSJ	2012	no	yes
DENV-4	KU509301.1	BR	2013	no	no
DENV-4	KY851722.1	BR	2012	no	no
DENV-4	KY851663.1	BR	2013	no	no
DENV-4	KY851664.1	BR	2013	no	no
DENV-4	KP703988.1	BRSPGU	2013	no	no
DENV-4	KP704065.1	BRSPGU	2013	no	yes
DENV-4	JQ513333.1	BRRRBV	2010	no	yes
DENV-4	JQ513341.1	BRRRBV	2010	no	no
DENV-4	JQ513330.1	BRRRBV	2010	no	no
DENV-4	JQ513332.1	BRRRBV	2010	no	no
DENV-4	JQ513331.1	BRRRBV	2010	no	yes
DENV-4	KY084518.1	BRRJ	2013	no	no
DENV-4	KY084517.1	BRRJ	2013	no	no
DENV-4	KY084516.1	BRRJ	2013	no	no
DENV-4	KP704105.1	BRSPGU	2013	no	no
DENV-4	KP704057.1	BRSPGU	2013	no	no
DENV-4	KP704000.1	BRSPGU	2013	no	no
DENV-4	KP703980.1	BRSPGU	2013	no	no
DENV-4	KP703953.1	BRSPGU	2013	no	no
DENV-4	KP703935.1	BRSPGU	2013	no	no
DENV-4	KP703921.1	BRSPGU	2013	no	no
DENV-4	KP703915.1	BRSPGU	2013	no	no
DENV-4	KP703885.1	BRSPGU	2013	no	no
DENV-4	KP704062.1	BRSPGU	2013	no	yes
DENV-4	KP638366.1	BRSPSJ	2012	no	no
DENV-4	KP638378.1	BRSPSJ	2014	no	no
DENV-4	KP638379.1	BRSPSJ	2012	no	no
DENV-4	KP188560.1	BRSPSJ	2012	no	no
DENV-4	KP188558.1	BRSPSJ	2012	no	no
DENV-4	KY084513.1	BRRJ	2012	no	yes
DENV-4	KY084515.1	BRRJ	2012	no	no
DENV-4	KY084511.1	BRRJ	2011	no	yes
DENV-4	KY084510.1	BRRJ	2011	no	no
DENV-4	JN848500.1	BR	2011	no	no
DENV-4	KY851712.1	BR	2012	no	no
DENV-4	KY851717.1	BR	2012	no	no
DENV-4	KY851751.1	BR	2013	no	no
DENV-4	JN848496.1	BR	2011	no	no
DENV-4	KP704116.1	BRSPGU	2013	no	no
DENV-4	JN559741.2	BRRRBV	2010	no	yes
DENV-4	KP704159.1	BRSPGU	2013	no	no
DENV-4	KP703882.1	BRSPGU	2013	no	no
DENV-4	KY851716.1	BR	2012	no	no
DENV-4	KY851715.1	BR	2012	no	no
DENV-4	KY851713.1	BR	2012	no	no
DENV-4	KY851714.1	BR	2012	no	no
DENV-4	KY851723.1	BR	2012	no	no
DENV-4	KY851720.1	BR	2012	no	no
DENV-4	KY851718.1	BR	2012	no	no
DENV-4	KY851719.1	BR	2012	no	no
DENV-4	KP638376.1	BRSPSJ	2013	no	no
DENV-4	KY851662.1	BR	2013	no	no
DENV-4	KP638373.1	BRSPSJ	2012	no	no
DENV-4	KP638358.1	BRSPSJ	2012	no	no
DENV-4	KP704145.1	BRSPGU	2013	no	no

Serotype	Accession code	Location	year	DAT-1	DAT-2
DENV-4	KP638371.1	BRSPSJ	2012	no	no
DENV-4	KP638370.1	BRSPSJ	2012	no	no
DENV-4	KP638364.1	BRSPSJ	2012	no	no
DENV-4	JN983813.1	BRRRBV	2010	no	yes
DENV-4	KP704126.1	BRSPGU	2013	no	no
DENV-4	KP704035.1	BRSPGU	2013	no	no
DENV-4	KP703981.1	BRSPGU	2013	no	no
DENV-4	KP703974.1	BRSPGU	2013	no	no
DENV-4	KP703963.1	BRSPGU	2013	no	no
DENV-4	KP703898.1	BRSPGU	2013	no	no
DENV-4	KP703891.1	BRSPGU	2013	no	no
DENV-4	KP704134.1	BRSPGU	2013	no	no
DENV-4	KP704103.1	BRSPGU	2013	no	no
DENV-4	KP704091.1	BRSPGU	2013	no	no
DENV-4	KP704086.1	BRSPGU	2013	no	no
DENV-4	KP704063.1	BRSPGU	2013	no	no
DENV-4	KP704044.1	BRSPGU	2013	no	no
DENV-4	KP704014.1	BRSPGU	2013	no	no
DENV-4	KP703993.1	BRSPGU	2013	no	no
DENV-4	KP703982.1	BRSPGU	2013	no	no
DENV-4	KP703961.1	BRSPGU	2013	no	no
DENV-4	KP703940.1	BRSPGU	2013	no	no
DENV-4	KP703879.1	BRSPGU	2013	no	no
DENV-4	KY084519.1	BRRJ	2013	no	yes
DENV-4	KP704210.1	BRSPGU	2013	yes	no
DENV-4	KY084521.1	BRRJ	2013	no	no
DENV-4	KY084514.1	BRRJ	2012	no	no
DENV-4	KP704043.1	BRSPGU	2013	no	no
DENV-4	KP704074.1	BRSPGU	2013	no	no
DENV-4	KP703946.1	BRSPGU	2013	no	no
DENV-4	KP704146.1	BRSPGU	2013	no	no
DENV-4	KP704081.1	BRSPGU	2013	no	no
DENV-4	KP704041.1	BRSPGU	2013	no	no
DENV-4	KP703965.1	BRSPGU	2013	no	no
DENV-4	KP703934.1	BRSPGU	2013	no	no
DENV-4	KP704143.1	BRSPGU	2013	no	no
DENV-4	KP704045.1	BRSPGU	2013	no	no
DENV-4	KP704056.1	BRSPGU	2013	no	yes
DENV-4	KP704206.1	BRSPGU	2013	no	no
DENV-4	KP703950.1	BRSPGU	2013	no	no
DENV-4	KP704108.1	BRSPGU	2013	no	no
DENV-4	KP704107.1	BRSPGU	2013	no	no
DENV-4	KP704084.1	BRSPGU	2013	no	no
DENV-4	KP704027.1	BRSPGU	2013	no	no
DENV-4	KP704020.1	BRSPGU	2013	no	no
DENV-4	KP704005.1	BRSPGU	2013	no	no
DENV-4	KP703969.1	BRSPGU	2013	no	no
DENV-4	KP703917.1	BRSPGU	2013	no	no
DENV-4	KP703880.1	BRSPGU	2013	no	no
DENV-4	KY851672.1	BR	2012	no	no
DENV-4	KP638360.1	BRSPSJ	2012	no	no
DENV-4	KP638362.1	BRSPSJ	2012	no	no
DENV-4	KY851754.1	BR	2013	no	no
DENV-4	KY851748.1	BR	2012	no	no
DENV-4	JN848497.1	BR	2011	no	no
DENV-4	KY851677.1	BR	2013	no	no
DENV-4	KP704019.1	BRSPGU	2013	no	no
DENV-4	KY851673.1	BR	2012	no	no
DENV-4	KY851671.1	BR	2012	no	no
DENV-4	KP704178.1	BRSPGU	2013	no	no

Serotype	Accession code	Location	year	DAT-1	DAT-2
DENV-4	KP704171.1	BRSPGU	2013	no	no
DENV-4	KY851750.1	BR	2013	no	no
DENV-4	JN848499.1	BR	2011	no	no
DENV-4	JN848498.1	BR	2011	no	no
DENV-4	JN092553.1	BR	2011	no	no
DENV-4	KP704205.1	BRSPGU	2013	no	no
DENV-4	KP704193.1	BRSPGU	2013	no	no
DENV-4	KP704192.1	BRSPGU	2013	no	no
DENV-4	KP704188.1	BRSPGU	2013	no	no
DENV-4	KP704187.1	BRSPGU	2013	no	no
DENV-4	KP704182.1	BRSPGU	2013	no	no
DENV-4	KP704181.1	BRSPGU	2013	no	no
DENV-4	KP704141.1	BRSPGU	2013	no	no
DENV-4	KP704118.1	BRSPGU	2013	no	no
DENV-4	KP704102.1	BRSPGU	2013	no	no
DENV-4	KP704070.1	BRSPGU	2013	no	no
DENV-4	KP704068.1	BRSPGU	2013	no	no
DENV-4	KP704066.1	BRSPGU	2013	no	no
DENV-4	KP704064.1	BRSPGU	2013	no	no
DENV-4	KP704054.1	BRSPGU	2013	no	no
DENV-4	KP704040.1	BRSPGU	2013	no	no
DENV-4	KP704033.1	BRSPGU	2013	no	no
DENV-4	KP704012.1	BRSPGU	2013	no	no
DENV-4	KP704003.1	BRSPGU	2013	no	no
DENV-4	KP704001.1	BRSPGU	2013	no	no
DENV-4	KP703995.1	BRSPGU	2013	no	no
DENV-4	KP703976.1	BRSPGU	2013	no	no
DENV-4	KP703943.1	BRSPGU	2013	no	no
DENV-4	KP703939.1	BRSPGU	2013	no	no
DENV-4	KP703933.1	BRSPGU	2013	no	no
DENV-4	KP703925.1	BRSPGU	2013	no	no
DENV-4	KP703923.1	BRSPGU	2013	no	no
DENV-4	KP703908.1	BRSPGU	2013	no	no
DENV-4	KP703906.1	BRSPGU	2013	no	no
DENV-4	KP703897.1	BRSPGU	2013	no	no
DENV-4	KP703895.1	BRSPGU	2013	no	no
DENV-4	KP703888.1	BRSPGU	2013	no	no
DENV-4	KP703887.1	BRSPGU	2013	no	no
DENV-4	KP703878.1	BRSPGU	2013	no	no
DENV-4	KP703877.1	BRSPGU	2013	no	no
DENV-4	KP703875.1	BRSPGU	2013	no	no
DENV-4	KP703865.1	BRSPGU	2013	no	no
DENV-4	KP703864.1	BRSPGU	2013	no	no
DENV-4	KP638367.1	BRSPSJ	2012	no	no
DENV-4	KP638363.1	BRSPSJ	2012	no	no
DENV-4	KP638359.1	BRSPSJ	2012	no	no
DENV-4	KP638355.1	BRSPSJ	2011	no	no
DENV-4	KT794007.1	BRAMMA	2011	no	yes
DENV-4	JF804055.1	MX	2006	yes	no
DENV-4	GU586124.1	HN	2007	yes	no
DENV-4	DQ390326.1	GF	1995	yes	no
DENV-4	DQ390324.1	GF	1995	yes	no
DENV-4	JF262782.1	HT	1994	yes	no
DENV-4	DQ390327.1	GF	1994	yes	no
DENV-4	DQ390329.1	GF	1994	yes	no
DENV-4	DQ390325.1	GF	1994	no	no
DENV-4	DQ390322.1	GF	1993	no	no
DENV-4	KU728213.1	DM	2006	yes	no
DENV-4	DQ390328.1	GF	2004	yes	no
DENV-4	DQ390323.1	GF	2005	no	no

Serotype	Accession code	Location	year	DAT-1	DAT-2
DENV-4	DQ390319.1	MQ	2004	yes	no
DENV-4	HM171571.1	MX	2006	yes	no
DENV-4	KU728215.1	DM	2006	yes	no
DENV-4	KU728214.1	DM	2006	yes	no
DENV-4	DQ390321.1	MQ	2004	yes	no
DENV-4	GQ868582.1	CO	2001	no	no
DENV-4	GQ868581.1	CO	2001	no	no
DENV-4	GQ868580.1	CO	2001	no	no
DENV-4	KC009637.1	CO	2004	no	no
DENV-4	KC009633.1	CO	2001	no	no
DENV-4	GQ868579.1	CO	2001	no	no
DENV-4	KC009634.1	CO	2001	no	no
DENV-4	KC009638.1	CO	2001	yes	no
DENV-4	JN819409.1	VE	1998	yes	no
DENV-4	GQ139585.1	VE	2001	no	no
DENV-4	FJ639748.1	VE	2000	no	no
DENV-4	FJ639745.1	VE	1999	no	no
DENV-4	GQ139582.1	VE	1999	no	no
DENV-4	GQ139581.1	VE	1999	no	no
DENV-4	FJ639744.1	VE	1999	no	no
DENV-4	FJ639739.1	VE	1998	yes	no
DENV-4	FJ639742.1	VE	1999	no	no
DENV-4	GQ139580.1	VE	1998	no	no
DENV-4	GQ139579.1	VE	1998	no	no
DENV-4	JF804051.1	VE	2001	no	no
DENV-4	FJ639736.1	VE	1998	no	no
DENV-4	GQ139578.1	VE	1997	no	no
DENV-4	FJ639764.1	VE	2001	no	no
DENV-4	FJ639738.1	VE	1998	no	no
DENV-4	FJ850059.1	PR	1998	no	no
DENV-4	EU854296.1	PR	1998	no	no
DENV-4	FJ882601.1	PR	1999	yes	no
DENV-4	FJ639737.1	VE	1998	yes	no
DENV-4	FJ882599.1	PR	1999	no	no
DENV-4	FJ024424.1	PR	1998	no	no
DENV-4	JF804057.1	PR	1998	no	no
DENV-4	GQ199883.1	PR	1996	no	no
DENV-4	GQ199881.1	PR	1996	no	no
DENV-4	GQ199878.1	PR	1994	no	no
DENV-4	GQ199884.1	PR	1996	no	no
DENV-4	FJ882597.1	PR	1998	no	no
DENV-4	FJ882600.1	PR	1999	no	no
DENV-4	EU854295.1	PR	1986	no	no
DENV-4	EU854297.1	PR	1998	no	no
DENV-4	FJ882596.1	PR	1998	yes	no
DENV-4	FJ882595.1	PR	1998	no	no
DENV-4	GQ199882.1	PR	1996	no	no
DENV-4	FJ882598.1	PR	1998	no	no
DENV-4	GQ199885.1	PR	1996	no	no
DENV-4	FJ850058.1	PR	1996	no	no
Total of selected sequences				58	44

Location acronyms: Brazil (BR); Colombia (CO); Dominica (DM); Dominican Republic (DO); Ecuador (EC); French Guiana (GF); Honduras (HN); Haiti (HT); Indonesia (ID); Martinique (MQ); Mexico (MX); Malaysia (MY); Peru (PE); French Polynesia (PF); Puerto Rico (PR); Senegal (SN); Suriname (SR); Trinidad and Tobago (TT); Venezuela (VE); Virgin Islands (VG); Amazonas, Manaus (BRAMMA); Minas Gerais, Divinópolis (BRMGDV); Mato Grosso, Cuiabá (BRMTCU); Mato Grosso, Nossa Senhora do Livramento (BRMTNL); Mato Grosso, Poconé (BRMTPO); Mato Grosso, Várzea Grande (BRMTVG); Paraná, Cambé (BRPRCA); Rio de Janeiro, Rio de Janeiro (BRRJRJ); Roraima, Boa Vista (BRRRBV); São Paulo, Guarujá (BRSPGU); São Paulo, São José do Rio Preto (BRSPSJ).

TABLE II

Sequence selection before and after the downsampling process for DAT-1

	Before downsampling		After downsampling	
Country	Number of samples	Sampling time span	Number of samples	Sampling time span
BR	502	1982-2014	5	1982-2013
CO	28	1982-2015	5	1982-2013
DM	3	2006	3	2006
DO	1	1997	1	1997
EC	8	2000-2014	5	1999-2014
GF	8	1993-2005	5	1994-2004
HN	1	2007	1	2007
HT	2	1994-2014	2	1994-2014
ID	1	2009	1	2009
MQ	2	2004	2	2004
MX	2	2006	2	2006
MY	10	1997-1999	2	1997-1999
PE	26	2000-2010	5	2000-2008
PF	6	1983-1988	5	1984-1988
PR	44	1986-2013	5	1995-2012
SN	1	1981	1	1981
SR	1	2010	1	2010
TT	1	2000	1	2010
VE	57	1995-2008	5	1995-2007
VG	1	1994	1	1994
Total	705	1981-2015	58	1981-2014

Location acronyms: Brazil (Brazil); Colombia (CO); Dominica (DM); Dominican Republic (DO); Ecuador (EC); French Guiana (GF); Honduras (HN); Haiti (HT); Indonesia (ID); Martinique (MQ); Mexico (MX); Malaysia (MY); Peru (PE); French Polynesia (PF); Puerto Rico (PR); Senegal (SN); Suriname (SR); Trinidad and Tobago (TT); Venezuela (VE); Virgin Islands (VG).

TABLE III

Sequence selection before and after the downsampling process for DAT-2

Brazilian location (State)	Before downsampling		After downsampling	
	Number of samples	Sampling time span	Number of samples	Sampling time span
Boa vista (RR)	9	1982-2010	5	1982-2010
Rio de Janeiro (RJ)	12	2011-2013	5	2011-2013
Guarujá (SP)	354	2013	5	2013
São José do Rio Preto (SP)	43	2011-2013	5	2012-2013
Divinópolis (MG)	3	2013	3	2013
Cuiabá (MT)	14	2012	5	2012
Nossa Senhora do Livramento (MT)	2	2012	2	2012
Poconé (MT)	3	2012	3	2012
Várzea Grande (MT)	7	2012	5	2012
Cambé (PR)	1	2013	1	2013
Manaus (AM)	6	2011	5	2011
Total	454	1982-2013	44	1982-2013

Location acronyms: Amazonas (AM); Minas Gerais (MG); Mato Grosso (MT); Paraná (PR); Rio de Janeiro (RJ); Roraima (RR); São Paulo (SP).
